# Host-microbiota interactions: from holobiont theory to analysis

**DOI:** 10.1186/s40168-019-0619-4

**Published:** 2019-01-11

**Authors:** Jean-Christophe Simon, Julian R. Marchesi, Christophe Mougel, Marc-André Selosse

**Affiliations:** 1 UMR 1349, IGEPP (Institut de Génétique, Environnement et Protection des Plantes), INRA, Agrocampus Ouest, Université Rennes 1, Domaine de la Motte, 35653 Le Rheu Cedex, France; 2 Centre for Digestive and Gut Health, Imperial College London, London, W2 1NY UK; 3 School of Biosciences, Cardiff University, Cardiff, CF10 3AX UK; 4 Muséum National d’Histoire Naturelle, Institut de Systématique, Évolution, Biodiversité, ISYEB-UMR 7205-CNRS, MNHN, UPMC, EPHE, Sorbonne Universités, 57 Rue Cuvier-CP39, F-75005 Paris, France; 5 Faculty of Biology, University of Gdansk, Ul. Wita Stwosza 59, 80-308 Gdansk, Poland

**Keywords:** Host-microbiota interactions, Holobiont, Hologenome, Metagenomics, Symbiosis

## Abstract

In the recent years, the holobiont concept has emerged as a theoretical and experimental framework to study the interactions between hosts and their associated microbial communities in all types of ecosystems. The spread of this concept in many branches of biology results from the fairly recent realization of the ubiquitous nature of host-associated microbes and their central role in host biology, ecology, and evolution. Through this special series “Host-microbiota interactions: from holobiont theory to analysis,” we wanted to promote this field of research which has considerable implications for human health, food production, and ecosystem protection. In this preface, we highlight a collection of articles selected for this special issue that show, use, or debate the concept of holobiont to approach taxonomically and ecologically diverse organisms, from humans and plants to sponges and insects. We also identify some theoretical and methodological challenges and propose directions for future research on holobionts.

## Background

It is becoming increasingly clear that the development, growth, and health (in one word, all functions) of macroorganisms are influenced by the complex microbial communities they host that shape their ecology and evolution [1–3]. Biology is indeed undergoing a paradigm shift, where individual phenotypes are seen as a result of complex interactions resulting from the combined expression of the host and associated microbial genomes, leading to the popularization of notions of the holobiont and the hologenome [4]. Holobiont research is now an imperative across numerous fields of the life and medical sciences, including aspects of mathematics (bioinformatics, statistics, and modeling). This change has pushed the scientific community to launch the first International Conference on Holobionts in Paris, April 19–21, 2017 [5]. This conference was organized by the French National Network on Environmental Genomics (GDR GE) and metaprogramme Meta-omics and microbial ecosystems (MEM-INRA), with supports from three French institutions, namely the National Center for Scientific Research (CNRS), the National Institute for Agricultural Research (INRA), and the National Museum of Natural History (MNHN). This meeting brought together about 200 scientists from 20 nationalities, highlighting the worldwide interest and the success of this first edition. Following this international conference, we proposed to dedicate a special issue of Microbiome Journal to holobiont research and collected a selection of articles that show, use, or debate the concept of holobiont to approach taxonomically and ecologically diverse organisms from all types of ecosystems.

## The holobiont concept rooted in symbiosis research

**Fig. 1:**
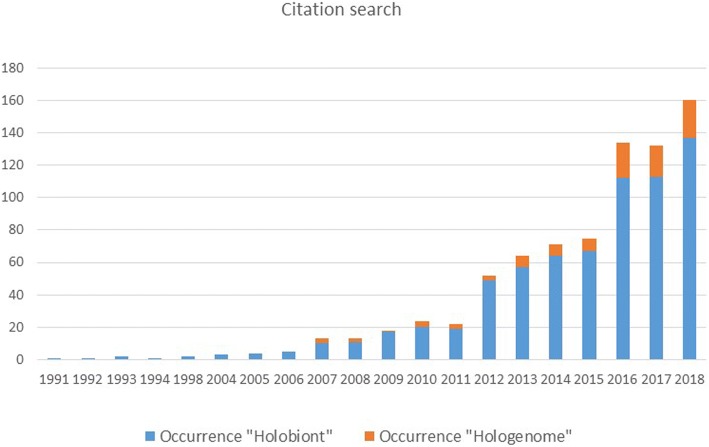
Occurrence of terms “Holobiont” (*N* = 695) and “Hologenome” (*N* = 102) in Web of Science Core Collection from 1991 to 2018

The term “holobiont” was first introduced in 1991 by Lynn Margulis [6] and initially referred to a simple biological entity involving a host and a single inherited symbiont. It was extended to define a host and its associated communities of microorganisms (also referred to as the microbiota which corresponds to the collection of microorganisms in interaction with their host and ranging from mutualistic to parasitic interactions). A host and its microbiota thus constitute a holobiont. This term is now widely used in different contexts and applies to virtually all metazoans, with current research focusing mainly on human, animal, and plant holobionts (Fig. 1). The term hologenome was introduced more recently in 2007 by Ilana Zilber-Rosenberg and Eugene Rosenberg [7, 8] to describe the sum of the host genome and associated microbial genomes, in other words, the collective genomes of a holobiont. For example, the human genome contains about 20,000 genes, but its hologenome contains > 33 million genes brought by its microbiota [9, 10].

## What does the holobiont concept bring new to biological and environmental sciences?

Per se, the holobiont concept is not new, as the roles and effects of microbes on animal and plant biology and evolution have long been recognized. The association between fungi and algae to form lichens was first reported by Anton De Bary who coined the term of symbiosis in 1879 [11]. In 1885, Frank coined the term mycorrhiza to describe beneficial fungi associated with tree roots [11]. The concept of rhizosphere and the role of rhizosphere microbiota on plant health have been subsequently highlighted in the early 1900s by Hiltner [12]. Buchner (1886–1978) made an impressive contribution to symbiosis research by describing many animal-symbiont associations, especially endocellular symbioses in insects, and by addressing the importance of symbiosis on their evolution [13]. What is actually new is the fairly recent realization of the ubiquitous nature of host-associated microbes and their central role in host biology, ecology, and evolution. There is no doubt that the development of molecular technics and NGS technologies has played an immense role in the recognition of microbes as key inhabitants of macroorganisms, and as players in biological and evolutionary processes, even if less studies address functional issues. Because many host-associated microbes are uncultivable outside their hosts, environmental genomics approaches have been successfully applied to unravel the diversity and roles of microbes in both model and non-model organisms and in all ecosystems whether terrestrial, marine, or aquatic, and spanning different types of host-microbiota associations from loose to tight.

This special issue contains a collection of 20 papers, covering a range of biological systems and taking different angles to offer the up-to-date state of the art in research, methodology, and application in the field of host-microbiota interactions. This collection nicely highlights the recent progress made in understanding the influence of microbes on their host’s evolution, ecology, and health and in developing the appropriate toolbox to better characterize and study the assembly process and the functioning of host-microbiota associations.

## New tools and methods to dissect holobiont components

The first issue regarding holobiont research is the characterization of microbiomes in hosts. While sequencing technologies are progressing rapidly both in the quantity and the length of sequences, allowing to generate large full-genome metagenomics datasets (the whole genome sequencing of the host and associated microbial communities), bioinformatics is currently challenged with the difficulty to sort reads from the different entities forming the holobiont and to explore microbial diversity at a fine resolution scale (i.e., strain-resolved metagenomics [14]). In this special issue, Guyomar and co-workers [15] present a methodological framework to characterize microbial diversity associated with aphid populations at species and intraspecies scales. Using this approach, on metagenomics read sets, these authors were able to reveal unsuspectedly high genomic diversity in different symbiont taxa both between and within their individual hosts. Meng and colleagues [16] propose a new method to separate the transcriptome of each member of the holobiont using metatranscriptomic datasets, allowing to access for the first time the whole functional diversity associated with the host and its microbiota. Young and co-workers [17] also used metatranscriptomics to characterize the microbiota associated with the wild grass *Holcus lanatus* and its changes in relation with soil types. In particular, they detected shifts in arbuscular mycorrhizal fungal communities according to phosphorus availability in the soil. Cregger and colleagues [18] characterize the respective effects of plant tissues and genotypes on microbial community structure in poplars. They found that microbial diversity varied primarily according to plant organs although fungal communities were also impacted by plant genotype.

## Assembly process to form a holobiont

Another key issue in holobiont research deals with the process of microbiota assembly and the mechanisms used by the host to control the source and maintenance of its multiple microbial partners. By experimentally disturbing the microbiota associated with *Daphnia* by an antibiotic treatment and inoculating germ-free individuals with the disturbed microbiota, Callens and co-workers [19] show that the antibiotic-induced disturbance strongly affects the holobiont assembly with consequences on host growth. Brener-Raffali and colleagues [20] explore on the coral species *Pocillopora damicornis* what factors determine microbial assemblies, involving algal and bacterial members. They showed that both genetic and environmental factors shape microbial composition and highlighted in particular the influence of sea temperature and its consequences on coral resilience in a context of global warming.

A primary factor determining the stability of microbiota in a host is its transmission pattern. There is a wide range of transmission modes for microbiota along a continuum from vertical to horizontal and environmental acquisition, but this important determinant of host-microbiota interactions is unknown in many systems. Vannier and co-workers [21] study the transmission of the microbiota associated with the plant *Glechoma hederacea* during vegetative propagation, an asexual reproduction mode very frequent in plants. They found that a significant proportion of the mother plants’ microbial communities were transmitted to their daughters through connections between individuals.

## Mechanisms and functions underlying host-microbiota interactions

What functions are delivered by the microbiota to the host and what are the resulting phenotypic and fitness effects are certainly among some of the most burning questions in the field of holobiont research. Additionally, the mechanisms underlying host-microbiota interactions are pivotal in our understanding of the functioning of holobionts. Van de Water and colleagues [22] review the recent advances made on host-microbe interactions in octocorals and illustrate some regulatory mechanisms of the microbiome by both the octocoral and its microbiota. They also reported the discovery of natural products with regulatory activities which may have potential biomedical applications. Sorek and co-workers [23] explore the area of the holobiont model in circadian rhythms in the corals and how their endosymbiotic algae’s biological clocks are integrated with the host. By taking examples in sponges, Pita and colleagues [24] stress how key functions, provided by holobionts to ecosystem functioning (such as biogeochemical cycling of key nutrients), can be affected by changes in microbiota composition. Ravanbakhsh and co-workers [25] review the influence of microbiota on plant ethylene signaling, an important pathway for triggering plant defense mechanisms. Guégan and colleagues [26] highlight the importance of the insect microbiota in the vectoring capacities of mosquitoes and discuss the potential offers by advanced knowledge on the mosquito holobiont for novel vector control strategies. Bredon and co-workers [27] tested whether the degradation of lignocellulose by terrestrial isopods involves their microbiota. By analyzing metagenomics and host transcriptomic datasets, these authors give evidence that both the host and its microbiota provide a complementary enzyme repertoire for lignocellulose degradation in this keystone soil invertebrate.

## Impacts of microbiota on host health

Microbiota is increasingly recognized for its major role on host health, and an important field application of holobiont research deals with the prevention and therapy of diseases based on treatments restoring altered microbiota. Van de Guchte and colleagues [28] address the importance of host-microbiota equilibrium in humans and discuss how perturbation of such homeostasis may lead to shifts from healthy to pre-disease and disease states. They propose that a large proportion of the Western lifestyle individuals are in a pre-disease state, which could explain the recent and explosive increase of inflammatory diseases and obesity in Western societies. Broberg and co-workers [29] report such shifts from healthy microbiota to pathobiota in the acute oak decline, a disease induced by a complex of microbes and which severely affects oak tree populations in Europe. Hassani and colleagues [30] point out the strong influence of microbe-microbe interactions within hosts on microbial community structure and underline their importance on the maintenance of host-microbial homeostasis.

## Evolutionary properties of holobionts

There is a hot debate in the community of evolutionary biologists on whether a holobiont should be considered as a unit of selection. The idea of holobionts as selection units was first introduced in 2008 by Zilber-Rosenberg and Rosenberg [8] as the hologenome theory of evolution. According to this theory, holobionts are viewed as superorganisms: interactions between members of the holobiont are mostly driven by cooperation and mutualism, and holobionts function as stable selectable entities [4]. For opponents to this theory, holobionts are rather unstable entities and viewed as ecosystems in which antagonism as well as mutualism and chance determine the interactions between members of the holobiont [2, 31, 32]. Ten years after, Rosenberg and Zilber-Rosenberg [33] revisit their theory in this special issue by refining their position on the specific evolutionary properties of holobionts. In a more consensual view, they conclude that rapid genomic changes in the microbiota facilitate the adaptation of holobionts to constantly changing environmental conditions.

In a longer evolutionary timescale, tight and persistent interactions between members of the holobiont may lead to genetic material exchange among this community entity. Sitaraman [34] reviews the recent discoveries made on horizontal gene transfer (HGT) within holobionts, focusing on the prokaryotic component of the human microbiota. This paper underlines the difficulties in detecting intra-prokaryotic HGT as well as in evaluating their rates and deciphering their mechanisms. By examining the impact of domestication on plant microbiome, Pérez-Jaramillo and co-workers [35] illustrate how human-induced pressure could drive the evolution of host-microbiota interactions in a not always desirable direction. Their meta-analysis indeed indicates a loss of plant-associated microbial diversity following domestication, with important consequences on resilient potential of crops to cope with environmental changes. Finally, Sevellec and colleagues [36] tested the hypothesis that microbiota could be a source and cause of host divergence ultimately leading to new species [37, 38]. By comparing the intestinal microbiota of pairs of whitefish species along a continuum of divergence, these authors did not detect parallelism between host and microbiota, suggesting that these two components forming the holobiont respond differently to selection and evolve each other with a large degree of autonomy.

## Conclusions

Collectively, the 20 articles of this series nicely exemplify the breadth and vitality of the research field studying holobionts, covering the different biological facets of host-microbiota interactions; the new developments to uncover their diversity, functioning, and evolution; and the applications for human health, food production, and ecosystem protection. This collection of papers highlights some future directions in the field of holobiont research but also points a number of challenges. Beyond metabarcoding, the role of microbiota in host adaptation and evolution needs to be addressed rigorously with both theoretical and experimental approaches, with a special attention paid to transmission patterns of microbiota components. Microbe-microbe interactions within holobionts should not be neglected as they participate in the host-microbiota equilibrium and should be approached in the framework of a multi-partner, diffuse coevolution. Studies integrating the taxonomic, genomic, and functional diversity associated with holobionts are still scarce, preventing a comprehensive understanding of holobiont assembly and functioning mechanisms. Omics-based advance methods have been crucial to study holobionts in all kinds of environments. However, these complex omic datasets represent bioinformatic challenges to integrate and disentangle the multiple components, interactions, and functions within holobionts. Also, the applications of holobiont research for enhancing host health or restoring perturbed ecosystems are still limited although their potential is increasingly recognized.

We hope this issue will stimulate more works on holobionts to meet these current and future challenges. Indeed, this field of research and the first Conference will soon find future steps in the 2nd International Conference on Holobionts (Montréal, Canada, 8–10 May 2019), the second of a promising series of meeting.
